# Ontogenetic Immaturity of Urachal Structures and Its Clinical Implications for Conservative Management in Children

**DOI:** 10.3390/children13060788

**Published:** 2026-06-05

**Authors:** Agata Maria Kawalec-Rutkowska, Anna Kawalec, Katarzyna Kiliś-Pstrusińska

**Affiliations:** 1 Department of Anatomy, Institute of Medical Sciences, University of Opole, Oleska 48, 45-052 Opole, Poland; 2 Clinical Department of Paediatric Nephrology, Faculty of Medicine, Wroclaw Medical University, Borowska 213, 50-556 Wroclaw, Poland; anna.kawalec@umw.edu.pl (A.K.); katarzyna.kilis-pstrusinska@umw.edu.pl (K.K.-P.)

**Keywords:** urachus, ontogeny, conservative treatment, child

## Abstract

**Highlights:**

**What are the main findings?**

- A subset of urachal abnormalities in children represents transient ontogenetic immaturity rather than permanent congenital pathology.
- Careful observation and longitudinal follow-up can be a safe alternative to early surgical intervention in selected asymptomatic or mildly symptomatic patients.

**What are the implications of the main findings?**

- Careful observation and longitudinal follow-up is a safe alternative to early surgical intervention in asymptomatic or mildly symptomatic cases.
- A developmental perspective helps avoid overtreatment and supports individualized care.

**Abstract:**

Abnormalities of the urachus detected in infancy and early childhood are often interpreted as persistent congenital abnormalities requiring surgical correction. However, growing clinical experience suggests that a proportion of these findings may reflect ontogenetic immaturity of the urachus rather than fixed pathological conditions. As a transient embryological structure, the urachus undergoes postnatal remodeling and involution, which may explain the spontaneous regression of urachal anomalies observed during follow-up. This paper proposes ontogenetic immaturity of the urachus as a biological substrate for the natural resolution of selected urachal changes, including cystic dilatation and incomplete obliteration. We discuss the developmental background of urachal maturation and emphasize the importance of distinguishing delayed involution from true structural pathology. The clinical implications of this concept are highlighted, with particular focus on the role of careful observation and longitudinal monitoring as an alternative to early surgical intervention in asymptomatic or mildly symptomatic patients. Recognizing ontogenetic immaturity as a reversible developmental state may help reduce overtreatment and support more individualized, developmentally informed management strategies. Integrating principles of developmental biology into clinical decision-making reinforces the value of conservative approaches in selected urachal conditions and under-scores the broader relevance of ontogeny in kidney and urinary tract disease.

## 1. Introduction

The urachus is a fibrous remnant of the allantois and the ventral portion of the cloaca, forming a cord-like structure that connects the urinary bladder to the anterior abdominal wall at the level of the umbilicus [1].

Pathologies of the urachus are extremely rare but must be recognized by urologists and pediatric surgeons [2]. Even though the urachus is a vestigial structure, it can give rise to significant conditions. Congenital anomalies occur when the urachus fails to fully regress, ranging from partially open forms—like urachal cysts, diverticula, or sinuses—to fully open forms, known as a patent urachus [1]. These abnormalities can present with a variety of clinical symptoms and are often identified on imaging studies, particularly if infection is present. A solid understanding of urachal embryology is therefore key to recognizing and interpreting them on imaging [1].

Urachal pathologies can be diagnosed in patients of any age, ranging from newborns and infants to adults [3,4]. Urachal remnants are found in approximately 1.03% of children, with a male-to-female ratio of 2:1, and in 92.5% of cases they are discovered incidentally [5]. Zenitani et al. reported that the prevalence of urachal remnants was greatest among children younger than 1 year and demonstrated a significant decreasing trend with increasing age, regardless of sex [6].

However, despite increasing recognition of urachal remnants in pediatric populations, their true clinical significance and natural history remain incompletely understood. There is still no clear consensus on whether these findings should be interpreted primarily as pathological entities requiring intervention or as transient developmental structures reflecting delayed involution. As a result, management strategies—particularly in asymptomatic or minimally symptomatic children—remain variable and controversial across clinical practice. Without proper management, patients face risks of recurrent symptoms, infections, or malignant transformation into adenocarcinoma [2,7]. This study aims to provide a developmental (ontogenetic) perspective on urachal abnormalities in children by analyzing the age-dependent prevalence of urachal remnants and evaluating patterns of spontaneous resolution in the context of conservative versus surgical management. It further explores whether delayed involution may underlie a subset of urachal findings and discusses the clinical implications of this process.

The study proposes ontogenetic immaturity of the urachus as a biological substrate for the natural resolution of selected urachal changes, including cystic dilatation and incomplete obliteration, with the aim of providing an exploratory synthesis of the currently available evidence and generating hypotheses to guide future clinical and research directions rather than establishing definitive clinical recommendations.

## 2. Materials and Methods

A literature search was performed using the PubMed; Web of Science, and Medline databases to identify publications addressing developmental aspects of the urachus in the pediatric population. The search covered the period from January 2016 to December 2025.

To improve search sensitivity, both free-text keywords and controlled vocabulary terms were applied. The search strategy included combinations of the following keywords and related terms: “urachus,” “urachal anomalies,” “urachal remnants,” “child,” “children,” “pediatric,” “infant,” “newborn,” “development,” “ontogeny,” “maturation,” “involution,” “spontaneous regression,” and “natural history.” In MEDLINE/PubMed, Medical Subject Headings (MeSH) were additionally incorporated, including terms such as “Urachus,” “Child,” “Infant,” and “Pediatrics,” combined with Boolean operators (AND/OR) to maximize retrieval of relevant studies.

The search was limited to studies published in English and involving human subjects. Titles and abstracts were screened to identify articles relevant to postnatal development, maturation, involution, or spontaneous regression of urachal structures in infancy and early childhood. Full texts of potentially eligible articles were reviewed to assess their relevance to the concept of ontogenetic immaturity of the urachus and its clinical implications.

Studies were included if they discussed developmental biology, natural history, or conservative management of urachal abnormalities in pediatric patients. Articles focusing exclusively on adult populations, malignant urachal disease, or purely surgical techniques without reference to developmental aspects were excluded.

The initial database search identified 55 records. After removal of duplicates, titles and abstracts were screened for eligibility. The majority of excluded articles focused on surgical techniques, adult urachal disease, or lacked discussion of developmental or ontogenetic aspects. After removal of duplicates, titles and abstracts were screened for relevance. Articles not addressing developmental or ontogenetic aspects of the urachus in pediatric populations were excluded. Six publications met the inclusion criteria and were included in the final qualitative synthesis.

A total of 6 publications meeting the inclusion criteria were identified and analyzed qualitatively. A summary of the six studies included in the review is presented in Appendix A, including authors, year of publication, title, and study type. Given the limited number and heterogeneity of available studies, a narrative synthesis was performed, emphasizing developmental mechanisms, patterns of spontaneous resolution, and implications for clinical management.

**Figure 1:**
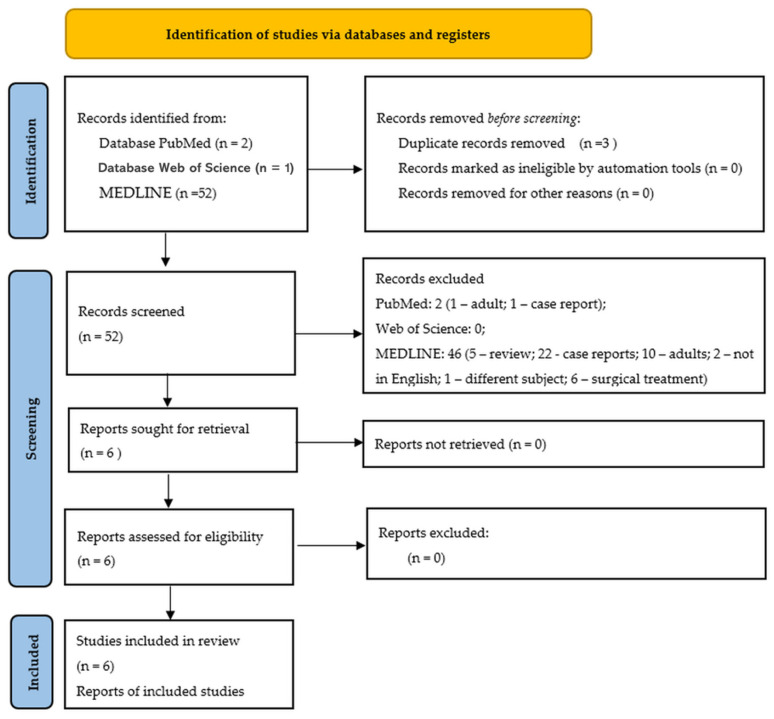
PRISMA flow diagram of study selection. Flow diagram showing the number of records identified through database searching, records after duplicates removed, screened records, full-text articles assessed for eligibility, and studies included in the final analysis, according to PRISMA recommendations.

The small number of included studies likely reflects both the rarity of focused research on urachal ontogeny in pediatric populations and the specificity of the inclusion criteria applied. Importantly, a broad search strategy was applied to minimize the risk of missing relevant studies. A flow diagram of the literature search and study selection process is presented in Figure 1.

## 3. Ontogeny

### 3.1. Urachal Ontogeny

During human embryogenesis the urachus originates from the allantois [8]. The allantois appears on the 16th day of embryonic development as a slender tubular structure originating from the yolk sac. It is continuous on one end with the ventral wall of the cloaca and on the other with the abdominal wall at the level of the umbilicus [9]. The urachus links the fetal bladder to the umbilicus, while the allantoic duct connects the umbilicus to the placenta; both structures normally undergo complete obliteration after birth [8].

By the third week of development, the cloacal region appears at the caudal end of the human embryo. The cloaca is subsequently divided by the urorectal septum into a ventral portion, which forms the urogenital sinus, and a dorsal portion, which develops into the hindgut. As mentioned above, the cloaca maintains a connection with the yolk sac through the allantois [5].

The urinary bladder initially develops from the ventral portion of the cloaca, known as the urogenital sinus, after separation of the cloaca into urinary and digestive portions. The bladder forms through mesenchymal–epithelial interactions between the endoderm of the urogenital sinus and surrounding mesodermal mesenchyme, with key signaling molecules involved in its development including Sonic Hedgehog (SHH), Transforming Growth Factor Beta (TGF-β), Bone Morphogenetic Protein 4 (BMP4), and Fibroblast Growth Factor Receptor 2 (FGFR2) [10]. As the embryo grows and the pelvis enlarges, the developing bladder becomes positioned more caudally in the pelvis [10,11].

At the fourth to eighth weeks of gestation, the bladder, formed from the urogenital sinus, differentiates and increases in size, while its connection with surrounding structures shifts as part of normal morphogenesis [10,11].

Before rupture of the cloacal membrane, the mesonephric duct drains into the cloaca and is thought to convey urine produced by the mesonephros from around the fifth week of gestation. During this period, a patent urachus may serve as a temporary urinary outlet. The cloacal membrane ruptures at the end of the seventh week of human embryonic development, establishing separate urogenital and anal openings following division of the cloaca by the urorectal septum [9].

The urachus itself represents the extension of the allantois into a tubular structure, becoming visible around the third week and linking the ventral cloaca to the yolk sac [5]. As the urinary bladder descends, its connection to the allantois—the urachus—gradually regresses. During this period, the allantoic duct and ventral cloacal remnants involute, and the bladder progressively adopts its definitive pelvic location [10,11]. This regression is typically completed between the 26th and 28th weeks of gestation, although the exact mechanism remains uncertain [5].

The bladder’s apparent “descent” reflects both the differential growth of the embryo and the contribution of surrounding tissues, rather than active migration. The growing bladder eventually lies entirely within the pelvis as development proceeds. This process of positional change and incorporation of surrounding ducts is part of normal urinary tract development, and deviations in the mechanisms directing these movements can contribute to congenital anomalies [10,11].

It is hypothesized that regression of the urachus may result from a slower growth rate of the urachus relative to the fetal body, accelerated fibrous proliferation of its wall, or hyperplasia/apoptosis of the urachal mucosa leading to lumen closure [5].

Once obliterated, the urachus persists as the median umbilical ligament [5]. The median umbilical ligament, a fibrous remnant of the urachus, is one of four fine ligaments located on the posterior aspect of the anterior abdominal wall. It runs vertically on the lower abdominal wall between the umbilicus and bladder. The other fine ligaments located on the posterior aspect of the anterior abdominal wall include the round ligament of the liver, a pair of medial ligaments, and a pair of lateral ligaments. The median and medial umbilical ligaments are enclosed by the parietal peritoneum, forming the median and medial umbilical folds on the posterior surface of the anterior abdominal wall [8].

The median umbilical ligament, together with the round ligament of the liver and the paired medial ligaments, converges at the umbilicus, reflecting their origin from structures within the umbilical cord: the round ligament from the umbilical vein, the medial ligaments from the umbilical arteries, and the median ligament from the urachus [8].

If regression of the urachus is incomplete, however, it may remain as a sinus, cyst, diverticulum, or a completely patent urachus [5].

The hypothesis of incomplete involution of the urachus includes failure of obliteration of the allantoic lumen and defective regression of embryologic tissues. The exact timing of physiological urachal lumen obliteration in healthy individuals remains unknown [9]. The precise timing of urachal closure remains a subject of debate, with previous studies suggesting it occurs between the 10th and 20th weeks post conception (WPC) [12,13,14]. Nevertheless, specific data indicating the exact gestational week at which the urachal lumen becomes obliterated are lacking. A limited fetal cohort demonstrated that fetuses younger than 16 WPC exhibited a patent urachal lumen with a cross-sectional area of approximately 18,000 μm^2^, whereas all fetuses at 17 WPC or older demonstrated complete obliteration of the urachal lumen [9]. According to previous histological observations, urachal lumen obliteration appeared to occur around the 17th WPC in the analyzed fetal specimens; however, the precise timing of physiological closure remains uncertain due to limited available histological data [9].

By the 17th WPC, corresponding to the stage when the urachal lumen was obliterated, histological analysis revealed a reduction in smooth muscle content accompanied by an increase in type I collagen in the urachus of both male and female fetuses. Concurrently, the transitional epithelium was no longer observed in fetuses with an obliterated lumen, whereas in younger fetuses it was present along the entire urachal tract. These changes indicate structural remodeling that likely culminates in fibrotic tissue formation [9]. However, these findings originate from a limited histological cohort and should be interpreted cautiously until validated by larger developmental studies.

Collagen and elastic fibers are key components of the extracellular matrix associated with fibrotic processes. Under picrosirius staining, greenish fibers corresponding to type III collagen—typically newly synthesized collagen produced during muscle regression—were predominant in younger fetuses, suggesting active tissue remodeling. In contrast, fetuses with an obliterated lumen showed a predominance of type I collagen, confirming that substantial tissue reorganization occurs prior to urachal closure. Biochemical quantification further demonstrated an increase in total collagen content in older female fetuses; however, no significant correlation was observed between total collagen levels and gestational age in male fetuses [9].

Although elastic fibers play a role in fibrotic tissue formation, Pazos et al. did not detect them in the urachus at these gestational stages, implying that this component of the extracellular matrix may emerge only during the third trimester [9].

Urachal anomalies have been reported to occur more frequently in males than in females. One histological study, the sex-related structural differences were identified in the fetal urachus at comparable gestational ages. The most notable distinction involved the connective tissue component: female fetuses exhibited a significantly higher proportion of connective tissue compared to male fetuses. Additionally, a positive correlation between connective tissue content and gestational age was observed exclusively in female fetuses, indicating that connective tissue accumulation increases with fetal maturation in females but not in males [9]. These findings suggest possible sex-related differences in urachal connective tissue composition; however, their clinical relevance remains uncertain. Further structural analyses comparing connective tissue in male and female patients with urachal pathologies would be required to validate this hypothesis [9]. At present, evidence supporting sex-dependent mechanisms of urachal involution remains preliminary and observational.

The most widely accepted explanation is that the lumen of the allantois does not close completely during late gestation or shortly after birth, which can result in a fully patent urachus or partial persistence manifesting as urachal cysts, sinuses, or diverticula. Some researchers describe these anomalies more broadly as a defective regression of embryonic tissues rather than an active failure of closure, leading to congenital remnants along the path from bladder to umbilicus. Although these abnormalities are generally attributed to a developmental failure of normal embryogenesis, the precise molecular or genetic factors that cause involution to fail in certain individuals remain poorly understood and are an area of ongoing study [9,15].

**Figure 2:**
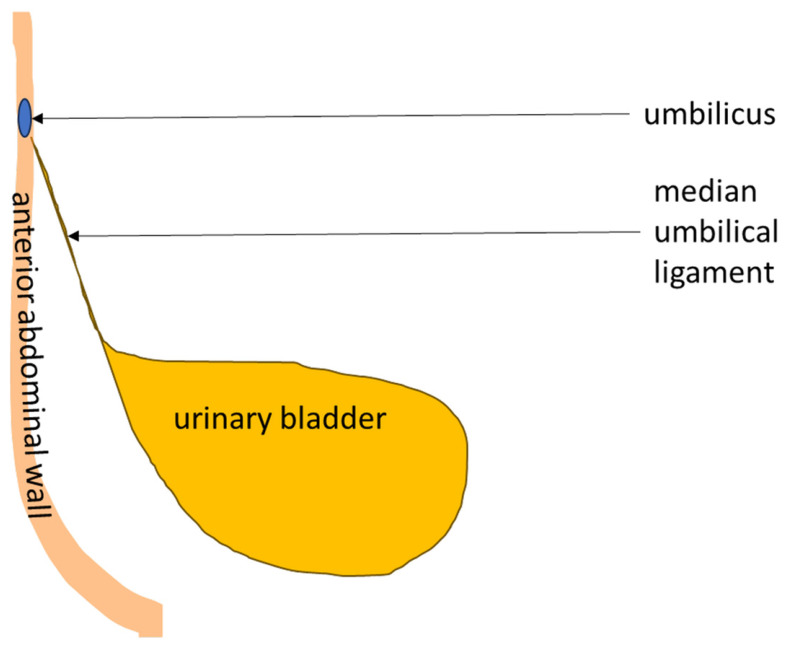
Schematic illustration demonstrating normal anatomy after physiological involution of the urachus, with transformation into the median umbilical ligament extending from the bladder dome to the umbilicus. The urinary bladder is shown in yellow, and the anterior abdominal wall is shown in orange. Digital illustration created by the author in Microsoft PowerPoint, based on anatomical references from Stoba C., Willital G. Atlas of Pediatric Surgery, 2008 [16].

Understanding the developmental timeline and mechanisms of urachal involution is clinically relevant, as delayed or incomplete obliteration may contribute to the persistence of asymptomatic urachal remnants observed in infancy and early childhood (Figure 2).

### 3.2. Urachal Anomalies

**Figure 3:**
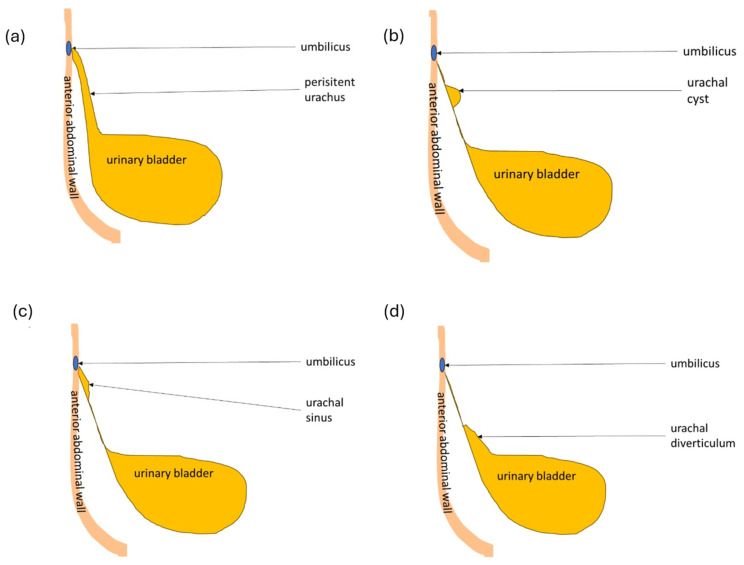
Schematic illustration of urachus anomalies: (**a**) patent urachus; (**b**) urachal cyst; (**c**) urachal sinus; (**d**) urachal diverticulum. The urinary bladder is shown in yellow, and the anterior abdominal wall is shown in orange. Digital illustration created by the author using Microsoft PowerPoint, based on anatomical references from Stoba C., Willital G. Atlas of Pediatric Surgery, 2008 [16].

Urachal anomalies are classified into four main types (Figure 3). Patent urachus and urachal cysts are the most common forms, accounting for approximately 50–52% and 30–60% of cases, respectively. Urachal sinus is less frequent, representing around 15% of cases, while urachal diverticulum is the rarest form, observed in only 3–5% of patients [5,17]. A patent urachus is defined as a persistent tubular communication between the urinary bladder and the umbilicus and is hypothesized to result from increased bladder pressure, such as that seen in obstructive uropathy [18,19]. A urachal cyst is a fluid-filled cavity located along the midline course of the urachus, most commonly situated near the dome of the bladder, and on ultrasound is typically visualized as a midline, fluid-filled sac [18]. An umbilical–urachal sinus is characterized by a blind-ending tract that opens at the umbilicus but does not communicate with the bladder, whereas a vesicourachal diverticulum is described as a localized outpouching arising from the bladder at the site of the urachal attachment [18].

#### Urachal Anomalies Laparoscopic Classification

Based on laparoscopic visualization of the urachal structure, Zenitani et al. proposed a classification of its anatomical variants into five types, taking into account the degree of obliteration and its relationship with the lateral umbilical ligaments [6]. Their observations demonstrated that postnatal urachal anatomy is highly variable and that the urachus does not invariably persist as a simple midline median umbilical ligament. In some individuals, the urachus deviated laterally or merged with adjacent ligamentous structures, suggesting that urachal involution and incorporation into the anterior abdominal wall may follow multiple anatomical patterns rather than a single uniform developmental pathway [6]. Complete or near-complete obliteration (type 0) represented the most common anatomical pattern, observed in 64.5% of patients [6]. The detailed laparoscopic classification is summarized in Table 1. Overall, currently available embryological and anatomical studies suggest that urachal involution is a gradual and variable developmental process; however, the underlying mechanisms and determinants of persistence remain incompletely understood.

## 4. Clinical Presentation and Diagnosis

### 4.1. Clinical Presentation

Except for a patent urachus, other urachal remnants are typically asymptomatic and are usually diagnosed incidentally [5,20]. However, complications associated with urachal anomalies often present with nonspecific clinical symptoms and may resemble other abdominal or pelvic conditions. Early recognition of potential complications, including infection and neoplasia, is essential to enable appropriate and timely management [18].

The most common clinical symptoms include umbilical discharge, granulation, and erythema in infants and abdominal pain in older children [20]. According to Orbatau et al., approximately half of analyzed pediatric cases were asymptomatic, while the most often complaints among symptomatic patients were abdominal pain (50%), umbilical discharge (37.5%), and a mass in the abdomen (12.5%) [21]. In contrast, Perez et al. reported that all the patients were symptomatic at presentation, with umbilical discharge (64%) and abdominal pain (24%) as main manifestations [17].

In neonates, a patent urachus may lead to continuous urinary leakage from the umbilicus. At birth, affected infants may present with a giant umbilical cord, as urine of low specific gravity is absorbed by Wharton’s jelly. Persistent wetting of the umbilical area after separation of the umbilical cord stump, along with umbilical granulation and erythema, is usually the main reason for parental concern [5,20]. Similarly, a urachal sinus may manifest as intermittent mucous secretion from its visible external opening, which can become purulent in the presence of infection [5]. Urachal remnants should be considered in the differential diagnosis of patients with delayed umbilical cord separation [22].

Older children may present with persistently wet or draining umbilicus, abdominal pain, or palpable mass. Noteworthy, there is a high risk of infection caused by Gram-positive skin flora or Gram-negative *Enterobacteriaceae*. In such situations, umbilical discharge becomes purulent. Inflammation of the urachal cyst results in continuous abdominal pain, erythema, or swelling, usually located below the umbilicus, and fever. In rare cases, intraperitoneal rupture of an infected urachal cyst may cause peritonitis [5,23]. Other symptoms of urachal remnants include recurrent urinary tract infections, hematuria, enuresis, and lower urinary tract symptoms (the feeling that the bladder is pulled towards the umbilicus during micturition, painful urination, and the sensation of residual urine) [5,17,20,24,25].

Malignant transformation of urachal remnants in children and adolescents is exceptionally rare, occurring predominantly in adults during the fifth and sixth decades of life. Urachal carcinoma accounts for less than 0.5–1% of all bladder malignancies, most commonly arising at the junction of the urachus and the bladder dome [5,26]. The pathogenesis is thought to involve malignant transformation of epithelial remnants, often associated with glandular metaplasia and possibly promoted by chronic inflammation or infection [5,27]. In a large pediatric cohort, most urachal anomalies contained epithelial components (72%), a factor postulated to be required for later adenocarcinoma development, but the absolute risk remains extremely low, with the number needed to excise to prevent a single case estimated at 5721 [27]. The most frequent presenting symptoms include hematuria, dysuria, abdominal pain, and occasionally a palpable lower abdominal or suprapubic mass. Mucous, purulent, or bloody umbilical discharge may also occur [26]. Only isolated cases of urachal adenocarcinoma have been reported in children and adolescents, including a 13-year-old girl and a 15-year-old boy presenting with hematuria, dysuria, abdominal pain, and progressively enlarging lower abdominal masses [26,28]. As noted by Lee, urachal carcinoma diagnosed below 30 years of age is extremely uncommon, with fewer than ten cases reported in the English-language literature [29].

### 4.2. Diagnosis

The diagnostic evaluation of suspected urachal anomalies begins with a thorough medical history and physical examination. In a proportion of cases, however, urachal remnants are identified incidentally during abdominal ultrasonography performed for unrelated indications [20]. The findings of Keçeli and Dönmez suggest that urachal remnants may be more common than historically assumed, particularly among children undergoing abdominal ultrasonography for other clinical reasons. In their retrospective observational study assessing 4836 children who underwent abdominal, urinary, or suprapubic ultrasonography over a one-year period, the overall prevalence of urachal remnants was approximately 2 per 1000 children, with urachal cysts representing the majority of cases [30].

#### 4.2.1. Proposed Diagnostic Algorithm for Urachal Anomalies

Based on the available literature, the diagnostic approach to suspected urachal anomalies can be summarized as a stepwise clinical algorithm.

In patients presenting with umbilical discharge, abdominal pain, a palpable infraumbilical mass, or recurrent urinary symptoms, initial evaluation should include a detailed medical history and physical examination. In neonates, persistent umbilical wetness or urine leakage from the umbilicus should raise immediate suspicion of a patent urachus.

In all suspected cases, ultrasonography is recommended as the first-line imaging modality due to its non-invasive nature, lack of radiation, and ability to visualize urachal anatomy and its relationship with the bladder dome and umbilicus. Ultrasound is generally sufficient to establish the diagnosis and to differentiate between patent urachus, urachal cyst, sinus, and diverticulum.

Prenatal detection of midline umbilical or infraumbilical cystic structures during routine obstetric ultrasonography should prompt postnatal follow-up, as many lesions may persist, regress, or become symptomatic after birth.

Cross-sectional imaging, including magnetic resonance imaging or computed tomography, should be reserved for selected cases in which ultrasound findings are inconclusive or complications such as infection, abscess formation, or suspected malignancy are present.

Voiding cystourethrography is indicated primarily in patients with suspected concomitant urinary tract abnormalities, including urinary tract infections, vesicoureteral reflux, or bladder outlet obstruction.

Overall, the diagnostic pathway should prioritize ultrasonography as the cornerstone of evaluation, with additional modalities used selectively based on clinical context and suspected complications. The diagnostic algorithm is shown in Table 2.

#### 4.2.2. Prenatal Ultrasonography

Prenatal ultrasonography serves as a valuable screening tool that enables early detection of urachal anomalies during routine fetal imaging. These abnormalities are most often identified in the late first or early second trimester [19,31]. The urachus typically appears as a tubular, fluid-filled structure extending between the anterosuperior surface of the bladder and the umbilicus, characterized by a hypoechoic wall and an anechoic lumen [19]. Urachal anomalies are visualized as midline cystic structures at the base of the umbilical cord or along the anterior abdominal wall [19,31]. A particularly characteristic feature in the first trimester is a “dumbbell-shaped” anechoic structure within the umbilical region, reflecting communication between the fetal bladder and an allantoic cyst [19,31]. Additional findings may include thickening of the umbilicus or the presence of an extra-abdominal cystic mass. In cases of a patent urachus, the cystic appearance may decrease as urine drains into the bladder [19].

#### 4.2.3. Urachal Anomaly Evolution in Prenatal Ultrasonography

Evidence from the literature shows that a patent urachus and urachal cyst may subsequently become undetectable in the third-trimester imaging, likely due to spontaneous in utero rupture [19,31,32,33]. The exact mechanism underlying rupture of a patent urachus remains unclear. One proposed explanation involves increased intravesical pressure, such as that seen in obstructive uropathy [33]. When rupture occurs in utero, a portion of the bladder may communicate with the amniotic cavity through the patent urachus. In such circumstances, bladder exstrophy must be excluded. The key elements in the differential diagnosis are visualization of a normally formed bladder and confirmation of an intact infraumbilical abdominal wall [19].

In a case report by Kwon et al., prenatal ultrasound at 13 weeks of gestation demonstrated a well-defined, 2.4 cm oval anechoic cyst at the proximal umbilical cord, with a visible connection to the fetal bladder. No abdominal wall defect or bowel herniation was observed, and the umbilical vessels were intact. The cyst enlarged to 5.4 cm at 17 weeks, then gradually decreased to 2.7 cm at 25 weeks, 1.8 cm at 32 weeks, and was undetectable at term. The bladder remained visible throughout gestation. Postnatally, a ruptured urachal cyst was observed at the neonate umbilicus and patent urachus was confirmed [19]. In Kwon’s additional review of 10 similar cases, the cysts disappeared at 26–30 weeks of gestation, presumably due to spontaneous rupture [19].

In a series reported by Umeda et al., five fetuses with urachus identified as an allantoic cyst were initially detected between 15 and 27 weeks of gestation. The cysts enlarged to a maximum diameter of 34–61 mm and subsequently underwent spontaneous rupture between 26 and 35 weeks. Postnatally, four infants were diagnosed with a patent urachus requiring surgical repair, while one infant had a urachal cyst managed conservatively [32].

Du et al. analyzed nine fetuses with patent urachus and allantoic cysts (PUAC), a rare embryologic anomaly typically presenting prenatally as an umbilical cystic mass communicating with the fetal bladder. PUAC were first detected between 12 and 16 weeks of gestation (median 13 weeks), with initial cyst diameters of 18–28 mm, fetal bladder diameters of 6–17 mm, and urachal widths of 1–2.5 mm. The cysts progressively enlarged during the second trimester, reaching maximum diameters of 26–53 mm between 19 and 29 weeks. Serial imaging demonstrated frequent spontaneous rupture, sometimes associated with protrusion of small umbilical masses through defects in the lower abdominal wall, followed by partial shrinkage or complete resolution of the cyst. More than half of the fetuses exhibited transient or persistent renal pelvic dilatation (5–6 mm), but no significant renal parenchymal abnormalities or lower urinary tract obstruction, and amniotic fluid volumes remained normal [31].

Bureau et al. reported a fetus with patent urachus associated with posterior urethral valves, suggesting that distal urinary obstruction may contribute to urachal patency. In their case, antenatal ultrasonography identified both allantoic cysts and patent urachus, subsequently found to be associated with posterior urethral valves (PUV). The cystic lesions were first detected in the early second trimester in the presence of a distended fetal bladder and a patent urachus. By 29 weeks’ gestation, the cysts had resolved spontaneously. Follow-up sonographic examinations demonstrated regular bladder emptying, and renal morphology remained normal throughout the pregnancy. The authors postulated that increased pressure within the urinary tract may have maintained urachal patency, promoted the formation of allantoic cysts, and ultimately led to their perforation. This decompression mechanism may have facilitated bladder emptying and mitigated the typical complications associated with PUV [33]. Notably, it was the first described case of a patent urachus associated with both allantoic cysts and posterior urethral valves in which the diagnosis was strongly suspected prenatally and subsequently confirmed after birth. This report underscores the clinical importance of careful detection and characterization of umbilical cord cysts during antenatal ultrasound evaluation. In the presence of an umbilical cord cyst communicating with the fetal bladder, obstructive uropathies should be considered in the differential diagnosis.

It is suggested that if the urachus persists, elevated bladder pressure could facilitate either its persistence or eventual rupture. However, the coexistence of urachal anomalies and obstructive uropathy is rare [19]. Taher et al. conducted a retrospective analysis over a 35-year period (1983–2018) to investigate the potential association between patent urachus and bladder outlet obstruction. Among 66 patients diagnosed with various urachal remnants, 16 were identified with a complete patent urachus. All patients presented clinically with umbilical discharge; in 10 cases the diagnosis was confirmed by micturating cystourethrography, and four patients had umbilical cord cysts detected on antenatal ultrasound [34]. Importantly, 25% of patients with patent urachus were found to have concomitant bladder outlet obstruction. The reported etiologies included posterior urethral atresia, congenital urethral hypoplasia, urethral atresia associated with prune belly syndrome, and sacrococcygeal teratoma. Additionally, vesicoureteral reflux was identified in 37% of cases, and among these, four patients had coexisting bladder outlet obstruction [34]. Although bladder outlet obstruction was not present in the majority of cases, this study highlights a clinically relevant association between patent urachus and obstructive uropathy. The authors recommend thorough postnatal evaluation, including ultrasound and cystography, to assess vesicoureteral reflux and potential obstruction, as well as renal function monitoring when reflux is detected. Their findings support the concept that, in a subset of patients, increased intravesical pressure secondary to obstruction may contribute to the persistence of urachal patency, even though the exact etiopathogenesis remains uncertain [34].

#### 4.2.4. Postnatal Imaging

Ultrasonography remains the preferred first-line modality for diagnosing urachal anomalies due to its ability to visualize their extra-peritoneal location, assess communication with the bladder, and avoid ionizing radiation [20,35,36]. It plays a central role in the initial evaluation of urachal remnants and is often sufficient for establishing the diagnosis and, in many patients, allows the characterization of the lesion [5,17,21,37]. On ultrasound, a patent urachus appears as a tubular, fluid-filled structure connecting the bladder dome to the umbilicus, with a hypoechoic wall and anechoic lumen. Umbilical–urachal sinuses are visualized as thickened hypoechoic tubular structures extending from the umbilicus without bladder communication, urachal cysts as thin-walled, midline lesions typically abutting the bladder dome with posterior acoustic enhancement, and vesico-urachal diverticula as extraluminal hypoechoic outpouchings from the bladder dome not connected to the umbilicus [35].

Ultrasound is generally an accurate diagnostic method, with sensitivity around 79% and positive predictive value of 83%, although false-negative findings are possible, particularly in small or atypical lesions [38]. It is also valuable for follow-up, enabling non-invasive monitoring of urachal remnants’ size, wall characteristics, and resolution [39].

Although ultrasonography is usually diagnostic, in some clinical situations it may be misleading, particularly when inflammation alters the expected anatomical relationships. Quinn reported a case of a boy whose symptoms strongly suggested acute appendicitis [40]. Both point-of-care ultrasound and a formal radiology-performed ultrasound were interpreted as consistent with acute appendicitis complicated by a periappendiceal abscess. The imaging findings were misleading due to inflammatory changes surrounding a midline urachal cyst, which caused secondary serositis involving the appendix and displacement of the cyst toward the right lower quadrant. This atypical anatomical and inflammatory presentation closely mimicked appendicitis on sonographic evaluation. Definitive diagnosis was established during emergent laparoscopy, which revealed an infected urachal cyst rather than appendiceal pathology [40].

In selected cases, additional cross-sectional imaging, such as magnetic resonance imaging or computed tomography, may be required to further delineate the anatomy and extent of the abnormality or to evaluate suspected complications such as infection or malignancy [5,17,20,21,25,35,37,41]. Voiding cystourethrography is usually reserved for patients with concomitant urinary tract infection or when abnormalities of the upper urinary tract are suspected [5,17,20,21,35,37].

## 5. Management

There is currently no clear consensus regarding the optimal management of urachal anomalies. Historically, surgical excision has been considered the standard approach, particularly in symptomatic patients [17,37], which is also reflected in the European Association of Urology (EAU) Paediatric Urology Guidelines [20]. Selected symptomatic patients might be managed conservatively initially. However, patients who do not show any clinical and radiological signs of regression should undergo surgical excision. According to retrospective data, surgical treatment was required in approximately 80% of symptomatic children with urachus remnants [17,23]. Noteworthy, the EAU guidelines acknowledge that in infants, especially those younger than six months, even symptomatic urachal remnants may be managed conservatively with observation and/or antibiotic therapy due to the high likelihood of spontaneous resolution. In cases of active infection, initial conservative treatment is recommended, with delayed elective surgery [20].

While some authors advocate routine surgical excision because of the presumed risk of malignant transformation, others emphasize that this risk is very low and raise concerns about overtreatment [17,27]. Accordingly, nonoperative management has increasingly been recognized as a safe and reasonable alternative, particularly in younger children [37].

### 5.1. Evidence Supporting Conservative Management

In recent years, an increasing number of studies have supported conservative, nonoperative management as a safe and effective alternative for pediatric urachal anomalies [21,37,42,43]. Dethlefs et al. demonstrated a clear shift in institutional practice toward nonoperative treatment, with the proportion of patients initially managed conservatively rising from 17.6% to 34.5% over time. In their cohort, conservative management based on clinical observation and ultrasonographic monitoring achieved a success rate of approximately 90%, with only a single patient requiring subsequent surgical intervention due to persistent drainage and fungal infection. Importantly, patients managed nonoperatively experienced fewer complications compared to those undergoing surgery, and no cases of abscess formation or sepsis were reported among individuals with known urachal remnants [37]. Similarly, Orbatu et al. retrospective analysis concluded that in majority of cases, follow-up of pediatric patients with urachal remnants can be performed conservatively [21]. In addition, Aylward et al. reported that surgical intervention in younger children was associated with an increased risk of reoperation, higher readmission rates, and prolonged hospital stay [43]. These findings support a more cautious, age-adapted approach, as conservative treatment has been shown to be effective, and deferring surgical excision of urachal remnants until after the age of 1 year may represent a preferable strategy in selected patients [43].

Zenitani et al. further investigated the prevalence and anatomic variants of urachal remnants in children using laparoscopic visualization. They analyzed medical records of 394 pediatric patients who underwent laparoscopic inguinal hernia repair and divided them into four age groups. Using laparoscopic assessment, the presence of a urachal remnant was confirmed in 140 children (35.5%). The prevalence was significantly higher in children younger than 1 year (63.2%) than in any other group, while no significant differences were observed among older age groups. In 42 cases (10.7%), the urachal remnant merged into the lateral umbilical ligament [6].

These results suggest that many urachal remnants observed in infancy may undergo spontaneous resolution, supporting nonoperative management of asymptomatic lesions, particularly in children under 1 year of age. This pattern is consistent with the concept of ontogenetic immaturity of urachal structures, as the underdeveloped nature of the urachus in infancy likely contributes to its high potential for spontaneous resolution, further reinforcing conservative management.

However, it should be emphasized that the available evidence is limited to small retrospective studies and observational series, which are subject to selection bias and heterogeneity, and therefore conclusions regarding the superiority or safety of conservative management should be interpreted with caution.

### 5.2. Surgical Management

Despite the efficacy of conservative management in selected patients, surgical intervention remains indicated for persistent urachal anomalies, recurrent or severe infections, cases unresponsive to antibiotic therapy, or abdominal pain causing acute abdomen [21]. Due to the potential risk of malignancy associated with epithelial-lined remnants, some authors suggest surgical excision even in asymptomatic patients [17]. However, remnants lacking epithelial lining are more likely to undergo spontaneous obliteration, and the actual risk of malignancy in asymptomatic children is extremely low [17,27]. Elective surgery is therefore generally not recommended for these patients [27].

#### 5.2.1. Surgical Techniques

When surgical intervention is indicated, several minimally invasive and open approaches may be used depending on the extent of the urachal remnant. The choice of technique is primarily guided by preoperative imaging and the anatomical extent of the lesion [25].

For lesions confined to the umbilical region, a trans-umbilical excision may be sufficient. When the remnant extends toward the bladder dome, laparoscopic-assisted approaches are commonly preferred, allowing complete excision with improved visualization and reduced invasiveness compared with open surgery [25,44]. In selected cases, purely laparoscopic or robotic-assisted techniques have also been described, reflecting the ongoing evolution of minimally invasive surgery in pediatric patients [45,46].

Overall, minimally invasive approaches are increasingly favored when technically feasible, while open surgery remains reserved for complex cases or associated anomalies requiring broader exploration [44].

Detailed descriptions of the surgical techniques reported in the included studies are provided in Appendix B.

#### 5.2.2. Special Considerations: Neonate with Patent Urachus with Allantoic Cyst (PUAC)

In cases of PUAC, surgical management cannot be postponed until 12 months of age due to the risk of complications and the need for early correction [31]. Du et al. reported that affected neonates often presented with a soft-tissue mass protruding from the umbilicus, which in some instances was covered by a thin membrane. The umbilical masses primarily consisted of prolapsed bladder tissue, occasionally involving intestinal tissue. In some neonates, a small amount of pale-yellow fluid was observed to discharge from the umbilicus during urination. All live-born infants underwent early surgical management, including excision of the urachal fistula, bladder repositioning, and umbilical reconstruction, with uneventful postoperative recovery and discharge approximately one week after surgery [31]. During long-term follow-up, clinical observations included abdominal discomfort and increased urinary frequency in some children. A subset of patients experienced urinary tract infections, and diagnostic evaluations revealed complications such as vesicoureteral reflux in certain cases. Follow-up assessments, including uroflowmetry and ultrasound, generally showed normal urinary flow rates, normal post-void residual volumes, and no significant structural abnormalities of the bladder or kidneys. In some children, cystic lesions in the bladder dome were noted; these lesions contained clear fluid, had well-defined borders, and remained stable in size and morphology during serial observations, consistent with pseudocysts rather than recurrent urachal remnants [31]. Overall, the study by Du et al. demonstrates that early surgical intervention for patent urachus with associated allantoic cysts is effective and safe, while long-term follow-up indicates generally favorable urinary function with occasional complications that can be identified and managed through ongoing monitoring.

### 5.3. Proposed Management Algorithm for Urachal Anomalies

The management of urachal anomalies can be structured into a stepwise clinical decision-making pathway based on patient age, symptom severity, presence of complications, and imaging findings. This algorithmic approach aims to integrate current evidence and facilitate standardized clinical decision-making between conservative and surgical treatment strategies, as summarized in Table 3.

Overall, the current body of evidence regarding the management of urachal anomalies in children is limited and largely based on retrospective, low-level studies; therefore, clinical recommendations remain non-standardized and should be individualized.

## 6. Conclusions

Taken together, these findings indicate that many urachal remnants in infants and young children represent manifestations of ontogenetic immaturity rather than fixed pathological lesions. The high prevalence of remnants in early infancy, combined with substantial rates of spontaneous resolution under conservative management, supports an age- and developmentally adapted approach. The marked decrease in prevalence after the first year of life (from 63.2% to approximately 28–32%) supports the hypothesis that many urachal remnants detected in infancy may represent transient developmental structures with a tendency toward spontaneous resolution [6]. Nonoperative management with clinical and ultrasonographic follow-up should be considered the first-line strategy for asymptomatic or minimally symptomatic patients, while surgical intervention should be reserved for persistent anomalies, recurrent or severe infections, or cases in which imaging demonstrates extensive involvement. Exceptions such as patent urachus with allantoic cysts require early operative correction. Histopathological evidence of urothelium in symptomatic or high-risk cases may justify surgery to prevent potential malignant transformation. High-risk cases may include patients with persistent urachal remnants that fail to regress during follow-up, recurrent inflammatory episodes, suspicious imaging findings such as solid components or marked wall thickening, extensive tissue involvement, or persistent symptoms despite conservative management. By aligning clinical decision-making with the underlying biology of urachal structures, this approach minimizes unnecessary interventions and associated morbidity while maintaining safety and efficacy, and ensures that follow-up identifies and manages any late complications or functional disturbances.

However, these conclusions should be interpreted with caution, as they are based on a limited number of available studies and primarily serve as an exploratory synthesis of the current evidence rather than definitive clinical recommendations. The findings should therefore be regarded as hypothesis-generating and supportive of clinical judgment, underscoring the need for further well-designed prospective studies to validate and expand upon these observations.

## Figures and Tables

**Table 1:** Laparoscopic classification of urachal anatomical variants according to Zenitani et al. [6].

Type	Description	Frequency
Type 0	Complete or near-complete obliteration of the urachus (<10 mm)	64.5%
Type 1	Midline fibrous cord extending from bladder apex to umbilicus without connection to lateral umbilical ligaments	23.1%
Type 2	Lateral deviation of the urachus with fusion to one lateral umbilical ligament	8.4%
Type 3	Midline urachus joining the lateral umbilical ligaments midway to the umbilicus	1.8%
Type 4	Lateral deviation and merging with one lateral umbilical ligament forming a single ligamentous structure	2.3%

**Table 2:** Proposed diagnostic algorithm for suspected urachal anomalies.

Step	Clinical Situation	Diagnostic Approach
1	Suspected urachal anomaly (umbilical discharge, infraumbilical pain/mass, recurrent UTI, neonatal umbilical wetness)	Clinical history and physical examination
2	Neonate with persistent umbilical drainage after cord separation	Clinical assessment + high suspicion of patent urachus
3	First-line imaging in all suspected cases	Ultrasonography (US)
4	Inconclusive ultrasound or suspected complications (infection, abscess, malignancy)	MRI or CT
5	Suspected urinary tract abnormalities (UTI, VUR, bladder outlet obstruction)	Voiding cystourethrography (VCUG)
6	Confirmed diagnosis	Clinical decision (conservative vs. surgical management)

**Table 3:** Management algorithm for urachal anomalies.

Clinical Situation	Management
Asymptomatic urachal remnant (incidental finding)	Observation ultrasound follow-up
Symptomatic <6 months (mild symptoms, no complications)	Conservative management ± antibiotics
Infection (urachal cyst/sinus/patent urachus)	Antibiotics → delayed surgery if persistent
Persistent symptoms or no regression on follow-up	Elective surgical excision
Acute abdomen/complicated infection/abscess	Urgent surgical intervention
PUAC (patent urachus with allantoic cyst)	Early surgical management

Arrows indicate the sequence of clinical decision-making steps.

## Data Availability

No new data were created or analyzed in this study.

## Abbreviations

The following abbreviations are used in this manuscript:

BMP4	Bone Morphogenetic Protein 4
BOO	bladder outlet obstruction
CT	computed tomography
FGFR2	Fibroblast Growth Factor Receptor 2
IQR	interquartile range
MCUG	micturating cystourethrography
MesH	Medical Subject Headings
PUAC	patent urachus with allantoic cyst
PUV	posterior urethral valves
SHH	Sonic Hedgehog
TGF-β	Transforming Growth Factor Beta
VUR	vesicoureteral reflux
WPC	weeks post conception

## Appendix A

**Table A1:** Characteristics of studies included in the review.

No.	Authors	Year	Title	Type of Study
1	Aylward P. et al. [43]	2020	Operative management of urachal remnants: An NSQIP based study of postoperative complications	Database-based retrospective study
2	Du L. et al. [31]	2025	From prenatal detection to postnatal evaluation: a retrospective observational ultrasound study of patent urachus with allantoic cyst	Retrospective observational ultrasound study
3	Keçeli A.M., Dönmez M.İ. [30]	2021	Are urachal remnants really rare in children? An observational study	Observational study
4	Nakagawa Y. et al. [25]	2022	Preoperative imaging contributes to pathologically complete resection of the urachal remnant by determining an appropriate surgical approach without unnecessary and excessive surgical invasion: a retrospective study	Retrospective study
5	Orbatu D., Alaygut D. [21]	2020	Evaluation and management of urachal remnants in children	Retrospective observational study
6	Zenitani M., Nose S., Oue T. [6]	2022	Prevalence of urachal remnants in children according to age and their anatomic variants	Retrospective observational study

## Appendix B

**Table A2:** Surgical techniques described in the included studies.

Technique	Description	Reference
Trans-umbilical (TU) excision	Circumumbilical incision with dissection of the urachal tract in the preperitoneal space and excision toward the bladder dome without entering the peritoneal cavity	Nakagawa et al. [25]
Laparoscopy-assisted trans-umbilical (LATU) approach	Laparoscopic guidance used to facilitate complete excision of urachal remnant extending toward the bladder apex while preserving bladder integrity	Nakagawa et al. [25]
Transumbilical reduced-port laparoscopic approach	Periumbilical incision with two transumbilical ports and an additional lateral port; dissection of urachus and excision at bladder dome with peritoneal closure	Aiyoshi et al. [45]
Robot-assisted laparoscopic excision (HIES technique)	Robot-assisted minimally invasive excision using hidden incision endoscopic surgery port configuration to achieve complete resection	Osumah et al. [46]
Open surgical approach (laparotomy)	Open excision of urachal remnant, with or without partial cystectomy depending on extent and associated anomalies	Taher et al. [44]
